# Modulation of heart rate by temporally patterned vagus nerve stimulation in the anesthetized dog

**DOI:** 10.14814/phy2.12689

**Published:** 2016-01-26

**Authors:** Paul B. Yoo, Haoran Liu, Juan G. Hincapie, Stephen B. Ruble, Jason J. Hamann, Warren M. Grill

**Affiliations:** ^1^ Department of Biomedical EngineeringDuke UniversityDurhamNorth Carolina; ^2^ Institute of Biomaterials and Biomedical EngineeringUniversity of TorontoTorontoCanada; ^3^ Department of Electrical and Computer EngineeringUniversity of TorontoTorontoCanada; ^4^ Cardiac Rhythm ManagementBoston Scientific CorporationSt PaulMinnesota; ^5^ Department of Electrical and Computer EngineeringDuke UniversityDurhamNorth Carolina; ^6^ Department of NeurobiologyDuke UniversityDurhamNorth Carolina; ^7^ Department of SurgeryDuke UniversityDurhamNorth Carolina

**Keywords:** Bradycardia, dog, heart failure, neuromodulation therapy, temporal patterned stimulation, vagus nerve stimulation

## Abstract

Despite current knowledge of the myriad physiological effects of vagus nerve stimulation (VNS) in various mammalian species (including humans), the impact of varying stimulation parameters on nerve recruitment and physiological responses is not well understood. We investigated nerve recruitment, cardiovascular responses, and skeletal muscle responses to different temporal patterns of VNS across 39 combinations of stimulation amplitude, frequency, and number of pulses per burst. Anesthetized dogs were implanted with stimulating and recording cuff electrodes around the cervical vagus nerve, whereas laryngeal electromyogram (EMG) and heart rate were recorded. In seven of eight dogs, VNS‐evoked bradycardia (defined as ≥10% decrease in heart rate) was achieved by applying stimuli at amplitudes equal to or greater than the threshold for activating slow B‐fibers. Temporally patterned VNS (minimum 5 pulses per burst) was sufficient to elicit bradycardia while reducing the concomitant activation of laryngeal muscles by more than 50%. Temporal patterns of VNS can be used to modulate heart rate while minimizing laryngeal motor fiber activation, and this is a novel approach to reduce the side effects produced by VNS.

## Introduction

Recent clinical studies suggest that vagus nerve stimulation (VNS) may be a safe (Schwartz et al. 2008) and potentially effective long‐term therapy for heart failure (De Ferrari et al. 2011; Premchand et al. 2014; Zannad et al. 2015). Selection of the therapeutic stimulation parameters are targeted to elicit a reduction in heart rate (bradycardia) and were translated from work in animals (Li et al. 2004). However, the same stimulation parameters that achieve bradycardia also result in various side effects that can undermine the long‐term therapeutic efficacy. As a consequence, the goal of this study was to investigate the effects of specific VNS parameters on selectively modulating changes in cardiac function.

The challenge of selectively modulating cardiac function by VNS is due to (1) the random distribution of nerve fibers within the predominantly unifasicular cervical vagus nerve; (2) the mix of efferent and afferent nerve fibers; and (3) the inverse electrical recruitment order of large‐to‐small diameter fibers by extracellular electrical stimulation. Vagal nerve fibers range from large myelinated A‐ and B‐type fibers, to unmyelinated C‐fibers (based on conduction velocity) (Hoffman and Kuntz 1957; Woodbury and Woodbury 1990), and recent work suggested further differentiation between fast and slowly conducting B‐type fibers (Yoo et al. 2013). Functionally, large myelinated A‐fibers provide efferent motor control of striated laryngeal muscles via the recurrent laryngeal nerve (Braund et al. 1988), whereas autonomic control of the other end‐organs is achieved by the much more abundant small B‐ and C‐type fibers (Evans and Murray 1954; Hoffman and Schnitzlein 1961; Satchell et al. 1982). However, the selective electrical recruitment of “therapeutically relevant” small diameter fibers (Woodbury and Woodbury 1990; Li et al. 2004) is challenging without concomitant activation of the low‐threshold large diameter fibers.

We investigated the feasibility of producing bradycardia, while minimizing the activation of laryngeal muscles, considered here as a side effect. This was achieved by systematically modifying the temporal pattern of VNS including the frequency and the number of pulses per burst. Nonsurvival experiments were conducted in anesthetized dogs, where both changes in heart rate and laryngeal muscle activity were used to quantify the differential effects of patterned VNS. Our results indicate that the specific type of nerve fiber (i.e., axon diameter) and the temporal pattern of activation both contribute to differential modulation of cardiac and skeletal muscle responses.

## Methods

All surgical and experimental protocols were approved by the Institutional Animal Care and Use Committee of Duke University. A total of 10 mongrel dogs (3 males, 7 females, 18–21 kg) were used in this study.

### Anesthesia

Animals were anesthetized with isoflurane (1–5%, inhalation) during all invasive surgical procedures, after which the anesthesia was switched to bolus i.v. injections of either a mixture of *α*‐chloralose and urethane (50 mg/kg and 500 mg/kg, 7 animals) or simply *α*‐chloralose (35–80 mg/kg, 3 animals). Although initial sedation/analgesia was achieved by three different methods – transdermal fentanyl patch (75 *μ*g/h) + propofol (4–6 mg/kg, bolus) in three animals, acepromazine (0.02–0.2 mg/kg, i.m. or s.q.) + propofol (4–6 mg/kg, bolus) in four animals, and only propofol (4–6 mg/kg, bolus) in three animals – these changes did not affect the electrical thresholds to excite vagal nerve fibers, nor did they alter the stimulation thresholds for achieving bradycardia. Heart rate (HR), blood pressure, jaw muscle tone, and paw withdrawal reflexes were periodically monitored to assess the depth of anesthesia. Any indication of responsiveness by the animal or a change in physiological status (e.g., changes in CO_2_ level, increase in blood pressure, or ocular reflex) resulted in an immediate bolus of anesthesia and/or analgesia (fentanyl, 10–15 *μ*g/h, i.v.). A continuous infusion of lactated ringer's solution (10 mL/kg/h, i.v.) was maintained throughout the experiment. At the end of the study, animals were euthanized with euthasol (0.2 mg/kg, i.v.).

### Instrumentation

The right cervical vagus nerve was accessed by a ventral incision. A bipolar, helical nerve electrode (Boston Scientific Corporation, St Paul, MN) was implanted on the cervical vagus nerve and connected to an external constant‐current stimulator (Pulsar 6 bp, FHC Incorporated). The stimulating pair of electrodes was configured as a rostral anode and caudal cathode (Agnew et al. 1989; Castoro et al. 2011; De Ferrari et al. 2014). A tripolar nerve cuff electrode (interelectrode distance = 5–7 mm, fabricated at Duke University) was implanted 7.5–12.5 cm distal to the helical electrode to record stimulation‐evoked compound nerve action potentials (CNAP). The vagus nerve at the recording site was desheathed (i.e., epineurium removed) prior to electrode implantation, which significantly improves the signal‐to‐noise ratio of the CNAP (Yoo et al. 2013). Bipolar, intramuscular wire electrodes were implanted in the laryngeal muscle to measure stimulation‐evoked efferent activity (EMG). All signals were filtered (CNAP = 100 Hz–30 kHz, EMG = 30 Hz–3 kHz) and amplified (CNAP = 10,000 gain, EMG = 1000 gain) using low‐noise amplifiers (SR560, Stanford Research Systems, Sunnyvale, CA). Standard surface electrodes were used to monitor the electrocardiogram (ECG) and arterial blood pressure was measured using a catheter in the femoral artery. All signals were recorded at a sampling rate of 20 kHz (PowerLab, ADInstruments Incorporated, Colorado Springs, CO).

### Experimental procedures

**Figure 1:**
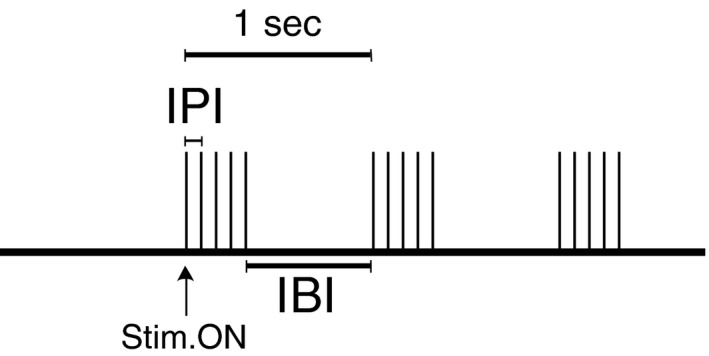
Temporally patterned vagus nerve stimulation (IPI = interpulse interval = frequency^−1^, IBI = interburst interval).

In eight animals, electrical recruitment of vagal nerve fibers was initially investigated by applying monophasic constant‐current pulses (pulsewidth = 300 *μ*sec, amplitude = 0.2–50 mA, frequency = 2 Hz, number of pulses = 20). Both the CNAP and EMG responses were used to determine the activation threshold of specific nerve fibers (myelinated A‐, fast B‐, slow B‐, and unmyelinated C‐fibers) and laryngeal muscles, respectively (Yoo et al. 2013). The threshold for eliciting bradycardia (BCT) was defined by the amplitude at which 20 sec of constant 20 Hz stimulation resulted in an approximately 10% decrease in heart rate. Based on this stimulation threshold, individual parameters were varied to achieve temporally modified patterns of stimulation (Fig. 1). The interpulse interval (IPI) and the number of pulses per burst were varied as indicated in Table 1. The interburst interval (IBI = 1 sec), pulse width (300 *μ*sec), and duration of pulse train (20 sec) remained constant. This stimulation protocol was repeated at two additional stimulation amplitudes determined by observation of the CNAP recording for each experiment: a value above BCT where B‐fiber recruitment was close to saturation (~1–2 mA above BCT); and at an amplitude below BCT where no recruitment of B‐fibers was observed.

**Table 1:** Summary of stimulation parameters. Each set of stimulation was repeated three times for each of the three stimulation intensities (below, at, and above bradycardia threshold amplitude)

Frequency	Temporal patterns
(Hz)	(Pulses per Burst)
10	2,5, continuous
20	1,2,5,10, continuous
50	2,5,10,20, continuous

In all experiments, we confirmed that the below‐BCT was greater than the amplitude for maximum activation of laryngeal muscles, and the amplitude corresponding to above‐BCT was lower than the threshold for activating unmyelinated C‐fibers. In a total of four dogs, the specific efferent/afferent VNS pathways responsible for modulating heart rate were confirmed by repeating electrical stimulation of either the proximal or distal stump after transecting the nerve trunk between the stimulating and recording electrodes.

### Data analysis

Stimulation‐evoked changes in heart rate were calculated by dividing the change in heart rate during stimulation by the resting heart rate. This value was expressed as a percent change in heart rate:

$$\text{Percent change in HR}=\left[\frac{\left({\text{HR}}_{\mathrm{stimulation}}-{\text{HR}}_{\mathrm{resting}}\right)}{{\text{HR}}_{\mathrm{resting}}}\times 100\right]\%$$

As the baseline heart rate varied throughout each experiment due to a variety of factors, the resting heart rate (HR_resting_) was defined as the average HR during the 20 sec prior to each stimulation trial (HR_stimulation_). Also, following each nerve stimulation trial a minimum of 30 sec was allowed to elapse so that any poststimulus changes in heart rate (e.g., transient tachycardia) returned to baseline. Transient poststimulation tachycardia was observed in all experiments, but not used to quantify changes in heart rate.

The stimulation‐evoked CNAP was quantified by calculating the rectified average of the response within specific poststimulus time windows (Castoro et al. 2011; Yoo et al. 2013). The laryngeal EMG was quantified by calculating the rectified average of the recorded signal within specific time windows (e.g., 5–20 msec, poststimulus) that accounted for both the distance travelled by action potentials and the conduction velocity of A‐fibers. In each experiment, the EMG response was normalized with respect to that measured during continuous 20 Hz stimulation at BCT.

Statistical analysis was with an ANOVA and, if appropriate, post hoc pair‐wise comparisons across stimulation parameters with Tukey–Kramer, with *P*‐values corrected for repeated comparisons.

## Results

### Thresholds for electrically recruiting vagus nerve fibers

**Figure 2:**
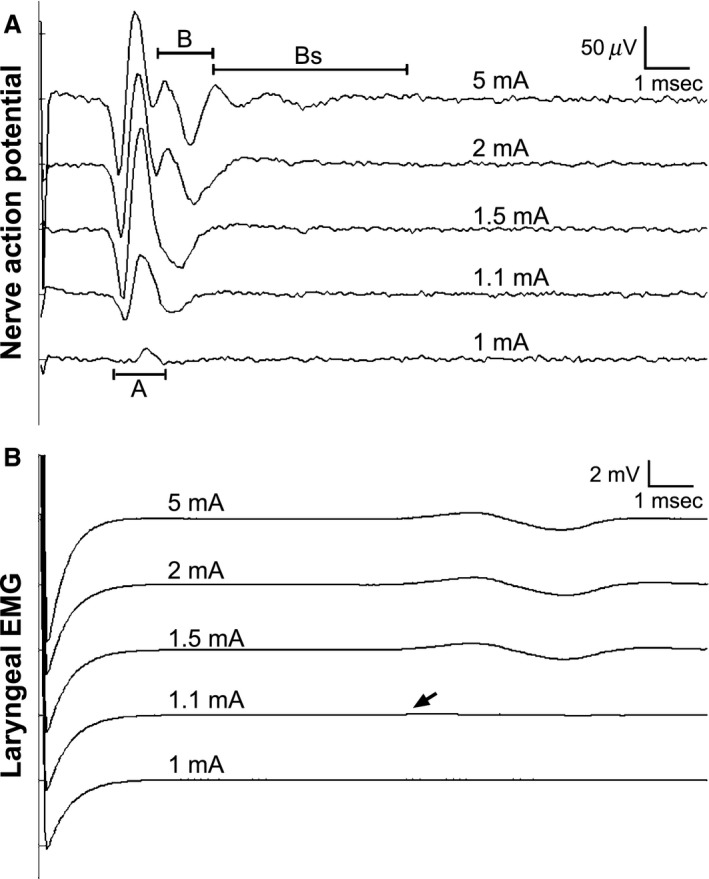
Sample data of electrically evoked signals from (A) the ipsilateral vagus nerve and (B) the laryngeal muscle. The recorded compound nerve action potential signal shows orderly recruitment of large diameter A fibers (1 –1.5 mA), B fibers (2 mA), and slow B‐fibers (5 mA, labeled as Bs). According to our previous study, this corresponded to conduction velocities of 38.8 ± 4.8 m/sec (A‐Fiber), 18.0 ± 4.7 m/sec (fast B‐fiber), and 10.5 ± 1.9 m/sec (slow B‐fiber). The laryngeal electromyogram shows the corresponding threshold activation of motor fibers at approximately 1.1 mA (arrow), and maximum muscle fiber recruitment at 1.5 mA.

Electrical stimulation of the cervical vagus nerve (amplitude = 0.1 up to 10 mA, 2 Hz) resulted in the recruitment of more rapidly conducting to more slowly conducing nerve fibers (i.e., larger‐to‐smaller diameters, Fig. 2A). Based on the poststimulus latencies of the components of the evoked CNAP, the activation threshold of each fiber type was determined in eight experiments: A‐fibers (0.52 ± 0.08 mA), fast B‐fibers (1.5 ± 0.2 mA), and slow B‐fibers (4.4 ± 0.5 mA). Electrical activation of large diameter fibers paralleled the laryngeal EMG, which typically reached maximum levels at stimulation amplitudes below 2 mA (Fig. 2B).

### Stimulation‐evoked changes in heart rate

**Figure 3:**
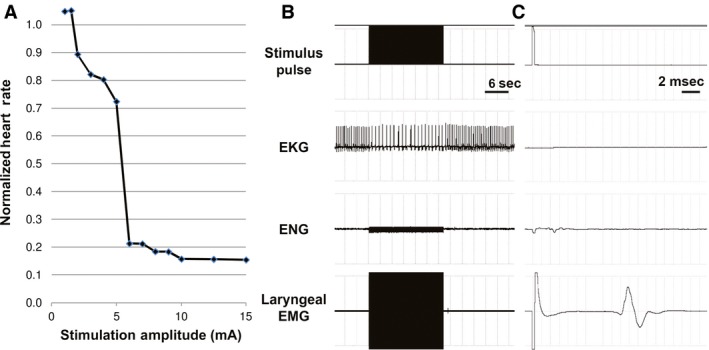
Continuous electrical stimulation of the right vagus nerve in anesthetized dogs (duration = 20‐sec, frequency = 20 Hz). (A) Response curve obtained from a single experiment, where the normalized heart rate was plotted against the stimulation amplitude. The bradycardia threshold (BCT) was 2 mA. (B) Electrical stimulation at 4 mA resulted in significant bradycardia (electrocardiogram, EKG), and correspondingly evoked neural (ENG) and muscle (laryngeal EMG) responses. (C) Zoomed image of the first electrical pulse shows the short‐latency ENG (1.8 msec) and longer‐latency EMG (9.5 msec) signals, which were repeated throughout the 20 sec of stimulation. [abscissa units: |EKG| ≤ 2.5 V, |ENG| ≤7.5 mV, and |Laryngeal EMG| ≤7.5 mV].

**Figure 4:**
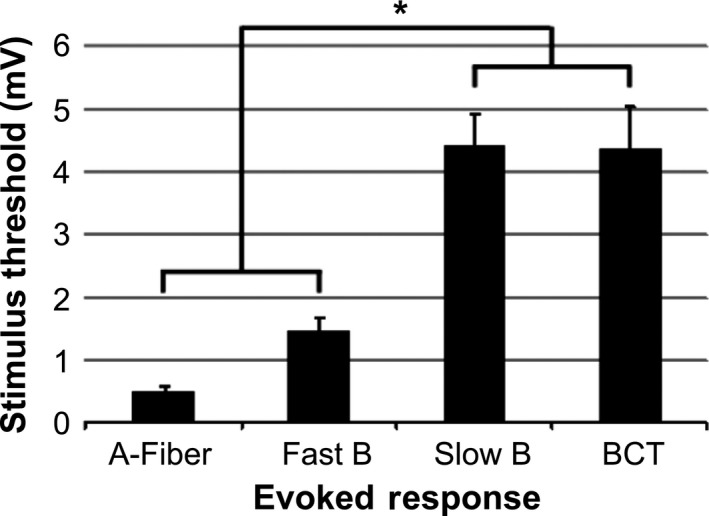
Activation thresholds of nerves fibers in the vagus nerve (*n* = 8 dogs) and bradycardia (BC) threshold. The bradycardia threshold coincided with the slow‐B fiber threshold. This relationship suggests that VNS‐evoked bradycardia is a result of the activation of this group of small, myelinated B‐fibers (*P ≤ 0.001).

The changes in heart rate produced by VNS were strongly dependent on the stimulation amplitude (Fig. 3A). As the amplitude was increased from 1 mA, stimulation‐evoked changes in heart rate transitioned from tachycardia (≤1.5 mA) to bradycardia (≥2 mA). Bradycardia occurred almost immediately with VNS and was sustained throughout the 20‐sec duration of stimulation (Fig. 3B). Each electrical pulse evoked a corresponding ENG (A, fast B, and slow B fibers) and laryngeal EMG response (Fig. 3C), where the magnitude of the evoked EMG was the same for all stimulation trials (i.e., maximum EMG achieved below BCT, Fig. 2B). In all experiments that exhibited VNS‐evoked bradycardia, a compensatory rebound in cardiac function was also observed, where transient tachycardia and increased blood pressure at the cessation of stimulation was followed by an eventual return to baseline. A comparison of the stimulation thresholds for activating the different vagal fiber types and the threshold for evoking bradycardia showed that there was no significant difference between slow B‐fibers and bradycardia threshold (BCT) (*P* = 0.94) (Fig. 4). Stimulation amplitudes used for all subsequent tests were based on BCT (4.4 ± 0.7 mA), below‐BCT (2.1 ± 0.5 mA), and above‐BCT (6.5 ± 0.8 mA).

#### Continuous vagus nerve stimulation: bradycardia versus laryngeal EMG

In seven of eight animals, continuous VNS (frequency = 20 Hz and amplitude = BCT) resulted in acute changes in heart rate. As shown in Table 2 (rightmost column), continuous VNS applied at BCT or above‐BCT amplitudes evoked reductions in heart rate (bradycardia), whereas VNS at below‐BCT amplitudes resulted in tachycardia. These responses were consistent across different stimulation frequencies (10–50 Hz). In three experiments, asystole was observed throughout the duration of VNS (amplitude = above‐BCT).

**Table 2:** Percent change in VNS‐evoked heart rate (# denotes *P* > 0.1; * denotes *P* < 0.05)

Freq(Hz)	Number of pulses				
	2	5	10	20	Continuous
Below BCT					
10	−0.96 ± 0.93	−0.82 ± 2.54			3.99 ± 3.32
20	−1.62 ± 1.43	−1.26 ± 1.39	−1.95 ± 2.62		7.48 ± 3.91
50	0.52 ± 1.90	0.35 ± 1.75	0.31 ± 3.32	5.58 ± 2.54	8.36 ± 3.28
BCT					
10	−3.37 ± 2.65	−4.48 ± 3.38			−6.77 ± 2.44^**#**^
20	−3.82 ± 2.37	−6.09 ± 2.94^**#**^	−7.62 ± 1.39^**#**^		−**11.1 ± 2.41**
50	−1.47 ± 2.64	−1.38 ± 2.23	−3.27 ± 1.32	−10.7 ± 4.11^**#**^	−15.6 ± 6.64^**#**^
Above BCT					
10	−0.88 ± 0.07	−2.23 ± 0.51			−11.9 ± 2.64^**#**^
20	−3.80 ± 2.08	−5.73 ± 2.12^**#**^	−16.7 ± 2.16*		−29.2 ± 3.11*
50	0.77 ± 4.49	−8.17 ± 2.04^**#**^	−17.5 ± 3.19^**#**^	−28.7 ± 14.2*	−57.9 ± 10.1*

Bold values correspond to the % change in heart rate and normalized laryngeal EMG activity achieved at the bradycardia threshold.

The stimulation amplitude was a significant factor in modulating cardiac function. Continuous VNS at BCT resulted in 5–16% (11.1 ± 2.41%) decreases in heart rate, which were further increased (10–60% bradycardia) at stimulation amplitudes above‐BCT. The correlation between the normalized heart rate (*y*) and the corresponding slow B‐fiber activity (*x*) at above‐BCT stimulation (linear regression, *y* = −0.9*x* + 1.8, *r* ^2^ = 0.38, *n* = 7) supported the negative chronotropic effects of these very small myelinated fibers. In contrast, continuous VNS at below‐BCT amplitudes resulted in 2–10% increases in heart rate. Statistical analysis showed that both the stimulation amplitude and frequency significantly affected heart rate independently (*P* < 0.001), but with significant interaction between the two variables (*P* < 0.001). For any given stimulation frequency (10 Hz, 20 Hz, or 50 Hz), the amplitude always had a significant effect on heart rate (*P* < 0.002); whereas, the stimulation frequency affected heart rate only at stimulation amplitudes of BCT (*P* < 0.05) or above‐BCT (*P* < 0.001). The stimulation frequency during below‐BCT VNS had limited effect on heart rate (*P* = 0.59). As shown in Table 2 (rightmost column), the average stimulation‐evoked reduction in heart rate achieved by continuous VNS at BCT (at 20 Hz) was not significantly different to responses evoked at 10 Hz at BCT, 50 Hz at BCT, and 10 Hz above‐BCT (#, *P* > 0.1). In contrast, the stimulation‐evoked reduction in heart rate evoked by 20 Hz at BCT were significantly different from those observed during below‐BCT stimulation (all frequencies, *P* < 0.001), and those applied at above‐BCT (20 Hz and 50 Hz, *, *P* < 0.05).

Given that all stimulation amplitudes used in this study were above the level of maximum laryngeal EMG activation, changes in the normalized EMG data (Table 3, rightmost column) were only dependent on the stimulation frequency (*P* < 0.01) and not the amplitude (*P* > 0.13). Compared to continuous nerve stimulation at 20 Hz BCT, VNS applied at 10 Hz and 50 Hz exhibited a 37% decrease and 78% increase in normalized EMG activity, respectively.

**Table 3:** Normalized laryngeal EMG (# denotes *P* > 0.1)

Freq(Hz)	Number of pulses				
	2	5	10	20	Continuous
Below BCT					
10	0.22 ± 0.10	0.35 ± 0.11			0.56 ± 0.12
20	0.22 ± 0.10	0.37 ± 0.09	0.64 ± 0.09		1.06 ± 0.15^#^
50	0.24 ± 0.15	0.30 ± 0.12	0.49 ± 0.16	0.82 ± 0.26^#^	1.78 ± 0.49^#^
BCT					
10	0.23 ± 0.09	0.36 ± 0.09			0.56 ± 0.03
20	0.23 ± 0.09	0.38 ± 0.08	0.59 ± 0.06		**1**
50	0.23 ± 0.08	0.34 ± 0.09	0.51 ± 0.10	0.88 ± 0.13^#^	1.69 ± 0.34
Above BCT					
10	0.27 ± 0.14	0.41 ± 0.11			0.62 ± 0.25
20	0.24 ± 0.13	0.29 ± 0.09	0.54 ± 0.19		0.84 ± 0.18^#^
50	0.26 ± 0.13	0.39 ± 0.15	0.55 ± 0.18	0.83 ± 0.28^#^	1.93 ± 0.55

Bold values correspond to the % change in heart rate and normalized laryngeal EMG activity achieved at the bradycardia threshold.

#### Temporally patterned vagus nerve stimulation: bradycardia versus laryngeal EMG

**Figure 5:**
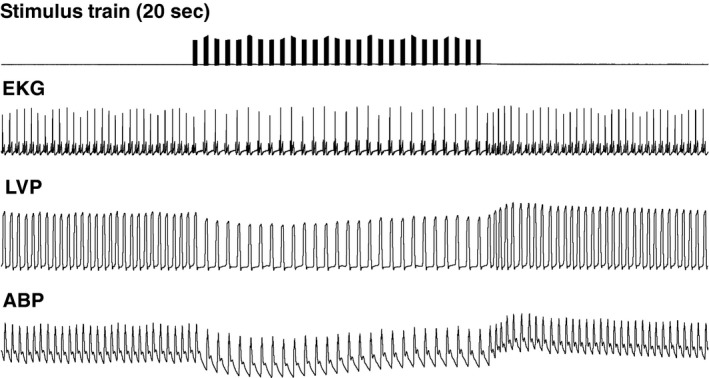
Characteristic cardiac effects of VNS (50 Hz, 20 pulses per burst, 5 mA – above bradycardia threshold). Stimulation pattern, electrocardiogram (EKG), and arterial blood pressure (ABP).

Temporally patterned VNS also elicited significant changes in heart rate that were dependent on additional stimulation parameters (Fig. 5). Both the stimulation amplitude and the number of pulses per burst were significant factors (*P* < 0.001). Compared to the level of bradycardia evoked by continuous 20 Hz VNS at BCT, temporally patterned VNS achieved comparable changes in heart rate with bursts of 5, 10, and 20 pulses that were applied at frequencies of 20 Hz or 50 Hz (Table 2, labeled as #). Bradycardia was elicited by VNS with as few as 5 pulses per burst when the stimulation amplitude was equal or greater than BCT. In fact, temporally patterned (bursting) stimulation at amplitudes above‐BCT, 10 pulses at 20 Hz or 20 pulses at 50 Hz, evoked greater changes in heart rate than continuous VNS (at BCT) at 20 Hz and 50 Hz. At stimulation amplitudes below‐BCT, temporally patterned VNS did not produce bradycardia, regardless of the number of pulses per burst and the stimulation frequency.

The temporally patterned VNS parameters that elicited bradycardia also resulted in markedly lower levels of laryngeal EMG activity as compared to continuous stimulation, regardless of the stimulation frequency (Table 3). VNS applied in bursts of 5 pulses and 10 pulses (e.g., 20 Hz) achieved normalized EMG activity that was approximately 75% and 41% lower, respectively, than the activity evoked by VNS at 20 Hz BCT. Temporally patterned VNS (20 Hz BCT at 5 pulses) generated laryngeal EMG activity that was approximately half of that evoked by continuous VNS (10 Hz BCT).

## Discussion

The objective of this study was to investigate a novel VNS paradigm designed to selectively decrease the baseline heart rate – a biomarker for heart failure therapy – in anesthetized dogs. Altering the temporal pattern of VNS parameters enabled differential modulation of cardiac function (bradycardia) and laryngeal activity (EMG activation). Periodic bursts of electrical pulses applied to the cervical vagus nerve, using as few as 5 pulses per second, resulted in specific chronotropic changes in cardiac function (10% bradycardia) while achieving substantial reductions in laryngeal muscle activation. The effects of altering the temporal patterns of VNS significantly expand our previous knowledge of how VNS‐mediated changes in heart rate can be sensitive to VNS parameters (e.g., amplitude, pulse width) (Yoo et al. 2011; Rousselet et al. 2014). Compared to the 2–10 sec of cyclical VNS that is currently used for treating heart failure patients (Schwartz and De Ferrari 2009; Zhang et al. 2009), our findings suggest that significantly shorter (0.25–0.5 sec) bursts of VNS can potentially achieve the same therapeutic outcomes. This approximately 10‐fold decrease in the overall activation of vagal nerve fibers (particularly the RLN, which is commonly linked to VNS‐related side‐effects) supports the potential therapeutic benefits of this novel VNS paradigm.

**Figure 6:**
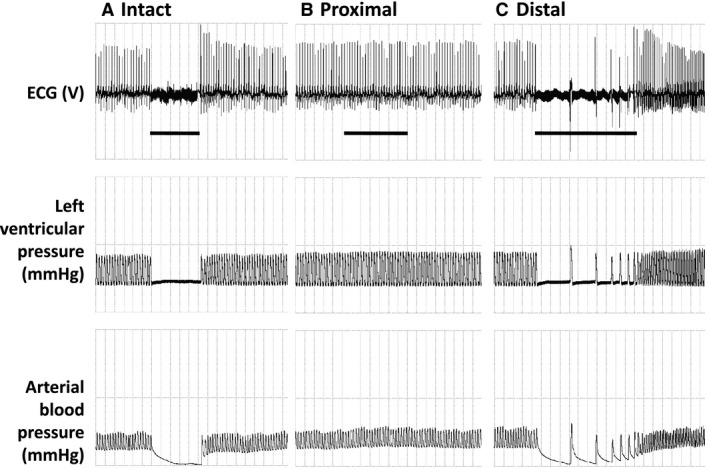
Continuous electrical stimulation of the (A) intact and (C) distal stump of the transected cervical vagus nerve both resulted in acute asystole (above BCT, 20 Hz). During stimulation‐evoked asystole, ventricular escape beats were commonly observed, as shown in panel C. In contrast, electrical stimulation of the (B) proximal stump of the transected cervical vagus nerve did not elicit any changes in cardiac function. [units per division: time (4 sec), ECG (1.5 V), left ventricular pressure (62.5 mmHg), arterial blood pressure (25 mmHg)].

Further, we confirmed that VNS‐evoked bradycardia requires the activation of slow B‐type fibers, which were previously characterized as slowly conducting (10.5 m/sec), small myelinated fibers (Yoo et al. 2013). In two experiments (Fig. 6), we also found that direct electrical stimulation of the transected distal stump of the right VN (and not the proximal end) could elicit bradycardia. These findings were consistent with previously published studies (Carlsten et al. 1957; Levy et al. 1969; Parker et al. 1984). As suggested by earlier work (Levy et al. 1972; Thompson et al. 1998; Buschman et al. 2006), the different anesthetic/analgesic agents used in this study did not affect the cardiac or skeletal muscle responses to VNS. We observed frequency‐dependent changes in heart rate during continuous VNS; whereas changes in heart rate during temporally patterned VNS were insensitive to stimulation frequency. With temporally patterned VNS, the heart rate was only responsive to changes the stimulation amplitude and/or the number of pulses applied per burst. These findings suggest a complex input‐output relationship producing a multidimensional dose–response curve for VNS, and underscore the critical role of VNS parameters in determining and controlling differentially physiological responses. It is also important to note that changes in parasympathetic and sympathetic tone, generated, for example, by baroreceptor reflex activity elicited during our VNS trials, either directly via stimulation of vagal afferents or indirectly by the evoked changes in BP and HR, may have contributed to variations in the trial by trial responses to VNS.

Low‐amplitude stimulation (below BCT) caused notable increases in heart rate. The literature attributes this to the electrical activation of sympathetic efferents located within the cervical vagus nerve trunk (Agostoni et al. 1957; Kimura et al. 1985), and histological analysis of the canine vagus nerve (data not shown) confirmed the presence of a small nerve fascicle traveling parallel to the vagus nerve, within the carotid neurovascular bundle. However, the low stimulation amplitudes at which we observed tachycardia are not consistent with the predicted activation threshold for small‐diameter sympathetic nerve fibers (A*δ* or unmyelinated C‐fiber). Based on the established recruitment properties of vagal nerve fibers in dogs (Anholt et al. 2011; Castoro et al. 2011; Yoo et al. 2013), the threshold for electrically activating these small nerve fibers (and thereby generating tachycardia) is expected to be much higher than BCT (≥9 mA). The fact that the magnitude of this response was limited to a 10% increase in heart rate – compared to the approximately 100% increase in heart rate during cardiac sympathetic nerve stimulation (Kimura et al. 1985; Onkka et al. 2013) – suggests that a very limited number of sympathetic fibers can be activated by stimulus pulses applied at or below 1 mA. Alternatively, low‐threshold afferents may reflexively modulate cardiac function, and this possibility is supported by our finding in two animals (data not shown) that stimulation of the proximal transected vagus nerve produced tachycardia (below‐BCT; 20–50 Hz), whereas stimulation of the distal nerve stump did not. Further work is needed to characterize this cardio‐excitatory reflex and also investigate potential effects on the efficacy of VNS therapies.

Among myriad potential therapies currently under investigation (Kirchner et al. 2000; Groves and Brown 2005; De Ferrari et al. 2011; Engineer et al. 2011), the recent use of VNS for improving long‐term morbidity in patients with heart failure has generated significant interest among clinicians and researchers (Schwartz and De Ferrari 2009; De Ferrari et al. 2011; Sabbah et al. 2011; Premchand et al. 2014; Zannad et al. 2015). However, the precise role of VNS‐evoked bradycardia in the treatment of heart failure remains unclear. From the earliest preclinical experiments involving a rodent cardiac infarction model (Li et al. 2004) to the canine ventricular over‐paced HF model (Zhang et al. 2009), VNS‐evoked bradycardia has served as the key physiological marker for achieving therapeutic outcomes. However, more recent studies in dogs (Hamann et al. 2013) and HF patients (De Ferrari et al. 2011; Premchand et al. 2014; Zannad et al. 2015) indicate that improvements in symptoms related to HF can be achieved in subjects that did not exhibit VNS‐evoked bradycardia. While such stimulation protocols (i.e., lower current amplitudes) were primarily a result of limited tolerance to VNS‐related side effects (Handforth et al. 1998; Sackeim et al. 2001; De Ferrari et al. 2011), these results suggest that bradycardia may not be a necessary condition for achieving HF therapy. As clinical trials proceed to investigate VNS therapy in HF patients (Hauptman et al. 2012; Dicarlo et al. 2013; De Ferrari et al. 2014), it may be interesting to investigate the long‐term therapeutic effects between approximately 50% of patients that achieved bradycardia (Schwartz et al. 2008) to those individuals that could not tolerate VNS amplitudes to cause bradycardia. It may also be important to investigate the potential therapeutic benefits of using temporally patterned VNS, when implemented at stimulation amplitudes above 1.42 ± 0.80 mA (Zannad et al. 2015). Further work is needed to clarify the precise vagal input(s) that mediate these therapeutic effects.

Peripheral nerve stimulation is a promising approach for long‐term treatment of a large range of chronic medical conditions (Birmingham et al. 2014) The anatomy of peripheral nerves, with multiple fascicles containing both motor and sensory fibers of varying diameters has motivated the development of a variety of approaches to achieve selective activation of targeted physiological responses. For example, unidirectional activation of nerve fibers can be achieved by asymmetric nerve cuff electrode designs (Sweeney and Mortimer 1986; Anholt et al. 2011) or high frequency nerve stimulation (Bhadra et al. 2006). Whereas selective activation of small‐diameter fibers (i.e., physiological recruitment order) or specific subsets of axons within a compound nerve trunk may be obtained by specific stimulus waveforms (Fang and Mortimer 1991) and/or novel electrode array designs (Branner et al. 2001; Lertmanorat and Durand 2004; Yoo et al. 2004; Kundu et al. 2014). The ability to activate components of the cervical vagus nerve in such a highly specific manner may provide the added benefit of avoiding side effects (Ramsay et al. 1994; Cristancho et al. 2011), which are a noted limitation of clinical VNS therapies. However, none of these technologies has been demonstrated effective in a clinical setting, and the bipolar helical nerve electrode is the only design that is currently approved by the FDA for VNS therapy.

Temporally patterned vagus nerve stimulation is a novel approach aimed at improving the selective activation of targeted physiological responses. In contrast to closed‐loop controlled implementations of VNS (Zhang et al. 2002; Tosato et al. 2006), we sought to achieve bradycardia by applying “predetermined paradigms” of electrical pulses in an open‐loop manner. The results of this study indicate that appropriately patterned stimulation can produce cardiac modulation with reduced side effects (less skeletal muscle activation) and therefore may increase the utility of VNS therapies. Temporally patterned VNS could offer an alternative approach to therapies for epilepsy and depression, but further testing is needed to determine the clinical efficacy of applying short bursts of high‐amplitude pulses in patients.

## Conflict of Interest

The authors disclose that JGH, JJH, and SBR are employees of Boston Scientific Corporation, and authors PBY, JGH, and WMG are co‐inventors of an issued US Patent.

## References

[phy212689-bib-0001] Agnew, W. F. , D. B. McCreery , T. G. Yuen , and L. A. Bullara . 1989 Histologic and physiologic evaluation of electrically stimulated peripheral nerve: considerations for the selection of parameters. Ann. Biomed. Eng. 17:39–60. 2537589 10.1007/BF02364272

[phy212689-bib-0002] Agostoni, E. , J. E. Chinnock , M. B. De Daly , and J. G. Murray . 1957 Functional and histological studies of the vagus nerve and its branches to the heart, lungs and abdominal viscera in the cat. J. Physiol. 135:182–205. 13398974 10.1113/jphysiol.1957.sp005703 PMC1358921

[phy212689-bib-0003] Anholt, T. A. , S. Ayal , and J. A. Goldberg . 2011 Recruitment and blocking properties of the CardioFit stimulation lead. J. Neural Eng. 8:034004. 21543838 10.1088/1741-2560/8/3/034004

[phy212689-bib-0004] Bhadra, N. , K. Kilgore , and K. J. Gustafson . 2006 High frequency electrical conduction block of the pudendal nerve. J. Neural Eng. 3:180–187. 16705274 10.1088/1741-2560/3/2/012 PMC3375816

[phy212689-bib-0005] Birmingham, K. , V. Gradinaru , P. Anikeeva , W. M. Grill , V. Pikov , B. McLaughlin , et al. 2014 Bioelectronic medicines: a research roadmap. Nat. Rev. Drug Discov. 13:399–400. 24875080 10.1038/nrd4351

[phy212689-bib-0006] Branner, A. , R. B. Stein , and R. A. Normann . 2001 Selective stimulation of cat sciatic nerve using an array of varying‐length microelectrodes. J. Neurophysiol. 85:1585–1594. 11287482 10.1152/jn.2001.85.4.1585

[phy212689-bib-0007] Braund, K. G. , J. E. Steiss , A. E. Marshall , J. R. Mehta , M. Toivio‐Kinnucan , and K. A. Amling . 1988 Morphologic and morphometric studies of the vagus and recurrent laryngeal nerves in clinically normal adult dogs. Am. J. Vet. Res. 49:2111–2116. 3239849

[phy212689-bib-0008] Buschman, H. P. , C. J. Storm , D. J. Duncker , P. D. Verdouw , H. E. van der Aa , and P. van der Kemp . 2006 Heart rate control via vagus nerve stimulation. Neuromodulation 9:214–220. 22151709 10.1111/j.1525-1403.2006.00062.x

[phy212689-bib-0009] Carlsten, A. , B. Folkow , and C. A. Hamberger . 1957 Cardiovascular effects of direct vagal stimulation in man. Acta Physiol. Scand. 41:68–76. 13497759 10.1111/j.1748-1716.1957.tb01510.x

[phy212689-bib-0010] Castoro, M. A. , P. B. Yoo , J. G. Hincapie , J. J. Hamann , S. B. Ruble , P. D. Wolf , et al. 2011 Excitation properties of the right cervical vagus nerve in adult dogs. Exp. Neurol. 227:62–68. 20851118 10.1016/j.expneurol.2010.09.011

[phy212689-bib-0011] Cristancho, P. , M. A. Cristancho , G. H. Baltuch , M. E. Thase , and J. P. O'Reardon . 2011 Effectiveness and safety of vagus nerve stimulation for severe treatment‐resistant major depression in clinical practice after FDA approval: outcomes at 1 year. J. Clin. Psychiatry 72:1376–1382. 21295002 10.4088/JCP.09m05888blu

[phy212689-bib-0012] De Ferrari, G. M. , H. J. Crijns , M. Borggrefe , G. Milasinovic , J. Smid , M. Zabel , et al. 2011 Chronic vagus nerve stimulation: a new and promising therapeutic approach for chronic heart failure. Eur. Heart J. 32:847–855. 21030409 10.1093/eurheartj/ehq391

[phy212689-bib-0013] De Ferrari, G. M. , A. E. Tuinenburg , S. Ruble , J. Brugada , H. Klein , C. Butter , et al. 2014 Rationale and study design of the NEuroCardiac TherApy foR Heart Failure Study: NECTAR‐HF. Eur. J. Heart Fail. 16:692–699. 24846173 10.1002/ejhf.80 PMC4288987

[phy212689-bib-0014] Dicarlo, L. , I. Libbus , B. Amurthur , B. H. Kenknight , and I. S. Anand . 2013 Autonomic regulation therapy for the improvement of left ventricular function and heart failure symptoms: the ANTHEM‐HF study. J. Card. Fail. 19:655–660. 24054343 10.1016/j.cardfail.2013.07.002

[phy212689-bib-0015] Engineer, N. D. , J. R. Riley , J. D. Seale , W. A. Vrana , J. A. Shetake , S. P. Sudanagunta , et al. 2011 Reversing pathological neural activity using targeted plasticity. Nature 470:101–104. 21228773 10.1038/nature09656 PMC3295231

[phy212689-bib-0016] Evans, D. H. , and J. G. Murray . 1954 Histological and functional studies on the fibre composition of the vagus nerve of the rabbit. J. Anat. 88:320–337. 13192020 PMC1244678

[phy212689-bib-0017] Fang, Z. P. , and J. T. Mortimer . 1991 Selective activation of small motor axons by quasi‐trapezoidal current pulses. IEEE Trans. Biomed. Eng. 38:168–174. 2066126 10.1109/10.76383

[phy212689-bib-0018] Groves, D. A. , and V. J. Brown . 2005 Vagal nerve stimulation: a review of its applications and potential mechanisms that mediate its clinical effects. Neurosci. Biobehav. Rev. 29:493–500. 15820552 10.1016/j.neubiorev.2005.01.004

[phy212689-bib-0019] Hamann, J. J. , S. B. Ruble , C. Stolen , M. Wang , R. C. Gupta , S. Rastogi , et al. 2013 Vagus nerve stimulation improves left ventricular function in a canine model of chronic heart failure. Eur. J. Heart Fail. 15:1319–1326. 23883651 10.1093/eurjhf/hft118 PMC3895958

[phy212689-bib-0020] Handforth, A. , C. M. DeGiorgio , S. C. Schachter , B. M. Uthman , D. K. Naritoku , E. S. Tecoma , et al. 1998 Vagus nerve stimulation therapy for partial‐onset seizures: a randomized active‐control trial. Neurology 51:48–55. 9674777 10.1212/wnl.51.1.48

[phy212689-bib-0021] Hauptman, P. J. , P. J. Schwartz , M. R. Gold , M. Borggrefe , D. J. Van Veldhuisen , R. C. Starling , et al. 2012 Rationale and study design of the increase of vagal tone in heart failure study: INOVATE‐HF. Am. Heart J. 163:954–62 e1. 22709747 10.1016/j.ahj.2012.03.021

[phy212689-bib-0022] Hoffman, H. H. , and A. Kuntz . 1957 Vagus nerve components. Anat. Rec. 127:551–567. 13425014 10.1002/ar.1091270306

[phy212689-bib-0023] Hoffman, H. H. , and H. N. Schnitzlein . 1961 The numbers of nerve fibers in the vagus nerve of man. Anat. Rec. 139:429–435. 13963923 10.1002/ar.1091390312

[phy212689-bib-0024] Kimura, T. , W. Uchida , and S. Satoh . 1985 Predominance of postsynaptic mechanism in vagal suppression of sympathetic tachycardia in the dog. J. Pharmacol. Exp. Ther. 235:793–797. 3001277

[phy212689-bib-0025] Kirchner, A. , F. Birklein , H. Stefan , and H. O. Handwerker . 2000 Left vagus nerve stimulation suppresses experimentally induced pain. Neurology 55:1167–1171. 11071495 10.1212/wnl.55.8.1167

[phy212689-bib-0026] Kundu, A. , K. R. Harreby , K. Yoshida , T. Boretius , T. Stieglitz , and W. Jensen . 2014 Stimulation selectivity of the “thin‐film longitudinal intrafascicular electrode” (tfLIFE) and the “transverse intrafascicular multi‐channel electrode” (TIME) in the large nerve animal model. IEEE Trans. Neural Syst. Rehabil. Eng. 22:400–410. 23799699 10.1109/TNSRE.2013.2267936

[phy212689-bib-0027] Lertmanorat, Z. , and D. M. Durand . 2004 Extracellular voltage profile for reversing the recruitment order of peripheral nerve stimulation: a simulation study. J. Neural Eng. 1:202–211. 15876640 10.1088/1741-2560/1/4/003

[phy212689-bib-0028] Levy, M. N. , P. J. Martin , T. Lano , and H. Zieske . 1969 Paradoxical effect of vagus nerve stimulation on heart rate in dogs. Circ. Res. 25:303–314. 5822518 10.1161/01.res.25.3.303

[phy212689-bib-0029] Levy, M. N. , T. Iano , and H. Zieske . 1972 Effects of repetitive bursts of vagal activity on heart rate. Circ. Res. 30:186–195. 5061318 10.1161/01.res.30.2.186

[phy212689-bib-0030] Li, M. , C. Zheng , T. Sato , T. Kawada , M. Sugimachi , and K. Sunagawa . 2004 Vagal nerve stimulation markedly improves long‐term survival after chronic heart failure in rats. Circulation 109:120–124. 14662714 10.1161/01.CIR.0000105721.71640.DA

[phy212689-bib-0031] Onkka, P. , W. Maskoun , K. S. Rhee , J. Hellyer , J. Patel , J. Tan , et al. 2013 Sympathetic nerve fibers and ganglia in canine cervical vagus nerves: localization and quantitation. Heart Rhythm 10:585–591. 23246597 10.1016/j.hrthm.2012.12.015 PMC3758134

[phy212689-bib-0032] Parker, P. , B. G. Celler , E. K. Potter , and D. I. McCloskey . 1984 Vagal stimulation and cardiac slowing. J. Auton. Nerv. Syst. 11:226–231. 6491162 10.1016/0165-1838(84)90080-8

[phy212689-bib-0033] Premchand, R. K. , K. Sharma , S. Mittal , R. Monteiro , S. Dixit , I. Libbus , et al. 2014 autonomic regulation therapy via left or right cervical vagus nerve stimulation in patients with chronic heart failure: results of the ANTHEM‐HF trial. J. Card. Fail. 20:808–816. 25187002 10.1016/j.cardfail.2014.08.009

[phy212689-bib-0034] Ramsay, R. E. , B. M. Uthman , L. E. Augustinsson , A. R. Upton , D. Naritoku , J. Willis , et al. 1994 Vagus nerve stimulation for treatment of partial seizures: 2. Safety, side effects, and tolerability. First International Vagus Nerve Stimulation Study Group. Epilepsia 35:627–636. 8026409 10.1111/j.1528-1157.1994.tb02483.x

[phy212689-bib-0035] Rousselet, L. , V. Le Rolle , D. Ojeda , D. Guiraud , A. Hagege , A. Bel , et al. 2014 Influence of Vagus Nerve Stimulation parameters on chronotropism and inotropism in heart failure. Conf. Proc. IEEE Eng. Med. Biol. Soc. 2014:526–529. 25570012 10.1109/EMBC.2014.6943644

[phy212689-bib-0036] Sabbah, H. N. , I. Ilsar , A. Zaretsky , S. Rastogi , M. Wang , and R. C. Gupta . 2011 Vagus nerve stimulation in experimental heart failure. Heart Fail. Rev. 16:171–178. 21128115 10.1007/s10741-010-9209-z PMC3784341

[phy212689-bib-0037] Sackeim, H. A. , A. J. Rush , M. S. George , L. B. Marangell , M. M. Husain , Z. Nahas , et al. 2001 Vagus nerve stimulation (VNS) for treatment‐resistant depression: efficacy, side effects, and predictors of outcome. Neuropsychopharmacology 25:713–728. 11682255 10.1016/S0893-133X(01)00271-8

[phy212689-bib-0038] Satchell, P. M. , J. G. McLeod , B. Harper , and A. H. Goodman . 1982 Abnormalities in the vagus nerve in canine acrylamide neuropathy. J. Neurol. Neurosurg. Psychiatry 45:609–619. 6288880 10.1136/jnnp.45.7.609 PMC491476

[phy212689-bib-0039] Schwartz, P. J. , and G. M. De Ferrari . 2009 Vagal stimulation for heart failure: background and first in‐man study. Heart Rhythm 6(11 Suppl):S76–S81. 19880077 10.1016/j.hrthm.2009.08.012

[phy212689-bib-0040] Schwartz, P. J. , G. M. De Ferrari , A. Sanzo , M. Landolina , R. Rordorf , C. Raineri , et al. 2008 Long term vagal stimulation in patients with advanced heart failure: first experience in man. Eur. J. Heart Fail. 10:884–891. 18760668 10.1016/j.ejheart.2008.07.016

[phy212689-bib-0041] Sweeney, J. D. , and J. T. Mortimer . 1986 An asymmetric two electrode cuff for generation of unidirectionally propagated action potentials. IEEE Trans. Biomed. Eng. 33:541–549. 3013759 10.1109/TBME.1986.325818

[phy212689-bib-0042] Thompson, G. W. , J. M. Levett , S. M. Miller , M. R. Hill , W. G. Meffert , R. J. Kolata , et al. 1998 Bradycardia induced by intravascular versus direct stimulation of the vagus nerve. Ann. Thorac. Surg. 65:637–642. 9527187 10.1016/s0003-4975(97)01351-9

[phy212689-bib-0043] Tosato, M. , K. Yoshida , E. Toft , V. Nekrasas , and J. J. Struijk . 2006 Closed‐loop control of the heart rate by electrical stimulation of the vagus nerve. Med. Biol. Eng. Comput. 44:161–169. 16937157 10.1007/s11517-006-0037-1

[phy212689-bib-0044] Woodbury, D. M. , and J. W. Woodbury . 1990 Effects of vagal stimulation on experimentally induced seizures in rats. Epilepsia 31(Suppl. 2):S7–S19. 2226368 10.1111/j.1528-1157.1990.tb05852.x

[phy212689-bib-0045] Yoo, P. B. , M. Sahin , and D. M. Durand . 2004 Selective stimulation of the canine hypoglossal nerve using a multi‐contact cuff electrode. Ann. Biomed. Eng. 32:511–519. 15117024 10.1023/b:abme.0000019170.74375.fb

[phy212689-bib-0046] Yoo, P. B. , J. G. Hincapie , J. J. Hamann , S. B. Ruble , P. D. Wolf , and W. M. Grill . 2011 Selective control of physiological responses by temporally‐patterned electrical stimulation of the canine vagus nerve. Conf. Proc. IEEE Eng. Med. Biol. Soc. 2011:3107–3110. 22254997 10.1109/IEMBS.2011.6090848

[phy212689-bib-0047] Yoo, P. B. , N. B. Lubock , J. G. Hincapie , S. B. Ruble , J. J. Hamann , and W. M. Grill . 2013 High‐resolution measurement of electrically‐evoked vagus nerve activity in the anesthetized dog. J. Neural Eng. 10:026003. 23370017 10.1088/1741-2560/10/2/026003

[phy212689-bib-0048] Zannad, F. , G. M. De Ferrari , A. E. Tuinenburg , D. Wright , J. Brugada , C. Butter , et al. 2015 Chronic vagal stimulation for the treatment of low ejection fraction heart failure: results of the NEural Cardiac TherApy foR Heart Failure (NECTAR‐HF) randomized controlled trial. Eur. Heart J. 36:425–433. 25176942 10.1093/eurheartj/ehu345 PMC4328197

[phy212689-bib-0049] Zhang, Y. , K. A. Mowrey , S. Zhuang , D. W. Wallick , Z. B. Popovic , and T. N. Mazgalev . 2002 Optimal ventricular rate slowing during atrial fibrillation by feedback AV nodal‐selective vagal stimulation. Am. J. Physiol. Heart Circ. Physiol. 282:H1102–H1110. 11834509 10.1152/ajpheart.00738.2001

[phy212689-bib-0050] Zhang, Y. , Z. B. Popovic , S. Bibevski , I. Fakhry , D. A. Sica , D. R. Van Wagoner , et al. 2009 Chronic vagus nerve stimulation improves autonomic control and attenuates systemic inflammation and heart failure progression in a canine high‐rate pacing model. Circ. Heart Fail. 2:692–699. 19919995 10.1161/CIRCHEARTFAILURE.109.873968

